# Role of ferroptosis and immune infiltration in intervertebral disc degeneration: novel insights from bioinformatics analyses

**DOI:** 10.3389/fcell.2023.1170758

**Published:** 2023-09-06

**Authors:** Xiao-Wei Liu, Hao-Wei Xu, Yu-Yang Yi, Shu-Bao Zhang, Shan-Jin Wang

**Affiliations:** ^1^ Department of Spinal Surgery, Shanghai East Hospital, School of Medicine, Tongji University, Shanghai, China; ^2^ Department of Orthopedic, East Hospital, Ji’an Hospital, Jinggangshan University School of Medicine, Jiangxi, China

**Keywords:** intervertebral disc degeneration, weighted gene co-expression network analysis, immune infiltration, ferroptosis-related genes, bioinformatics analyses

## Abstract

**Background:** Intervertebral disc degeneration (IVDD), which contributes to stenosis of the spinal segment, commonly causes lower back pain. The process of IVDD degradation entails gradual structural adjustments accompanied by extreme transformations in metabolic homeostasis. However, the molecular and cellular mechanisms associated with IVDD are poorly understood.

**Methods:** The RNA-sequencing datasets GSE34095 and GSE56081 were obtained from the Gene Expression Omnibus (GEO) database. Ferroptosis-related differentially expressed genes (DEGs) were identified from these gene sets. The protein–protein interaction (PPI) network was established and visualized using the STRING database and Cytoscape software, and the key functional modules of ferroptosis-related genes were identified. Gene ontology (GO) and Kyoto Encyclopedia of Genes and Genomes (KEGG) pathway enrichment analyses were performed on the DEGs. Weighted gene co-expression network analysis (WGCNA), immune infiltration analysis in the GEO database, and other GSE series were used as validation datasets. The xCELL algorithm was performed to investigate the immune cell infiltration differences between the degenerated IVDD and control groups.

**Results:** The major genes involved in nucleus pulposus tissue immune infiltration and ferroptosis-related genes were mined by bioinformatics analysis. A total of 3,056 DEGs were obtained between the IVDD tissue and control groups. The DEGs were enriched in the cell cycle; apoptosis; necroptosis; and the PI3K-Akt, Hippo, and HIF-1 signaling pathways. PCR and Western blot techniques were utilized to confirm the differential ferroptosis-related genes. The results indicated that the protein expression levels of NCOA4 and PCBP1 were elevated, while the protein expression level of GPX4 was reduced in NPCs following IL-1β treatment. Our study has found that severe disc tissue degeneration leads to a noteworthy increase in the expression of CD8A in naive T cells, CCR7 in memory CD4^+^ cells, GZMB in natural killer (NK) cells, and CD163 and CD45 in macrophages.

**Conclusion:** Our data demonstrate that ferroptosis occurs in IVDD, suggesting that ferroptosis may also increase IVDD improvement by triggering immune infiltration. This work was conducted to further understand IVDD pathogenesis and identify new treatment strategies.

## 1 Introduction

Low back pain is a growing problem worldwide, with intervertebral disc degeneration (IVDD) being the primary degenerative condition associated with this issue. This has led to significant health problems and has placed a substantial financial burden on those affected (Livshits et al., 2011; Cieza et al., 2021). Nucleus pulposus (NP) dysfunction is considered to be the pivotal element in IVDD (Binch et al., 2021). The cellular properties of nucleus pulposus cells (NPCs) decrease as they become senescent, and the accumulation of these cells can lead to chronic inflammation and matrix degradation (Cherif et al., 2020; Lyu et al., 2021). Degeneration of intervertebral discs can result in the deterioration of the extracellular matrix and loss of hydrophilic matrix molecules. This alteration in disc biomechanics can lead to elevated inflammation levels and pain factors (Risbud and Shapiro, 2014; Zehra et al., 2022). Intervertebral disc degeneration is driven by various molecular mechanisms, including DNA replication errors; metabolic disturbances; inflammation; and loss of disc matrix, functional cells, and stem cells. The degeneration of the NP cells can trigger a cascade of chronic events, such as an imbalance between extracellular matrix synthesis and catabolism, leading to asymmetry (Le Maitre et al., 2007a; Wang et al., 2016; Lu et al., 2021; Yang et al., 2021). Extensive research has been conducted to determine the most effective treatment for IVDD. Early diagnosis and minimally invasive therapy are recommended for the initial stages of the disease. Inhibiting programmed cell death (PCD) and other forms of regulated cell death (RCD) has been studied as therapeutic objectives for many years, and this approach has become the primary method for treating early-to-mid-stage IVDD cases (D'Arcy, 2019; Ariga et al., 2001; Zhao et al., 2007; Erwin et al., 2015). Ferroptosis is a regulated form of cell death characterized by iron-dependent lipid peroxidation. This process leads to the accumulation of lethal levels of lipid peroxidation in cells (Peng et al., 2022). Ferroptosis has been implicated in various degenerative diseases, including Parkinson’s disease and Alzheimer’s disease. Recent studies have also suggested that ferroptosis may have a significant role in IVDD (Stockwell et al., 2017; Zhang X. et al., 2020). Deferoxamine, a ferroptosis inhibitor, has been shown to attenuate disc degenerative strategies in a rat model of IVDD, suggesting that ferroptosis may play a role in injury-induced disc degeneration (Yang et al., 2021).

Inflammatory cytokines and chemokines play a crucial role in recruiting immune cells to the intervertebral disc and its associated tissues. This recruitment is a critical step in the pathway that leads to pain production (Risbud and Shapiro, 2014; Khan et al., 2017; Francisco et al., 2022). Numerous studies have shown a potential link between ferroptosis and infection, inflammation, and tumors. Meanwhile, immune cells play an important role in the aforementioned processes (Wang and Lu, 2022). In the fight against human diseases, particularly inflammatory diseases, various immune cells such as neutrophils, T lymphocytes, B lymphocytes, macrophages, NK cells, and dendritic cells (DCs) play a crucial role. Additionally, these immune cells may be regulated by specific forms of cell death during different functional stages of their action. Recent studies have demonstrated the crucial role of ferroptosis in regulating immune cell function. Ferroptosis of immune cells can have a significant impact on their number and function, which in turn affects the self-immune response. Conversely, ferroptosis of non-immune cells can trigger the release of damage-associated molecular patterns, leading to immune cell responses (De Domenico et al., 2008; Chen X. et al., 2021). Despite its potential significance, the role of ferroptosis and immune cells in disc tissue degeneration has not been thoroughly studied. It is crucial to further investigate these factors to develop effective therapeutic approaches for IVDD without causing tissue damage.

This study aimed to identify the immune cell types and key genes associated with disc degeneration through gene expression matrices. We also investigated the relationship between ferroptosis and infiltrating immune cells to gain a better understanding of the molecular immune processes involved in IVDD development. Additionally, we conducted basic experiments to validate the bioinformatics-mined genes.

## 2 Materials and methods

### 2.1 Data source

The datasets used in this study were obtained from the GEO database, which can be found at https://www.ncbi.nlm.nih.gov/geo/. Specifically, we downloaded the gene expression profiles from two studies: GSE34095 and GSE56081 (Wan et al., 2014); GSE124272 (Wang et al., 2019); GSE41883, GSE27494, and GSE23130 (Gruber et al., 2012); and GSE70362 (Kazezian et al., 2015). The training set consisted of GSE34095 and GSE56081, while the validation set included GSE124272, GSE41883, GSE27494, GSE23130, and GSE70362. To merge the information sets, we utilized the R software package in SilicoMerging (Taminau et al., 2012), as well as the approach of Johnson et al., to eliminate the batch effect and subsequently obtain the matrix (Johnson et al., 2007).

### 2.2 Differentially expressed gene identification and functional enrichment analysis

The limma package in R was utilized to analyze differential gene expression in the GSE34095 and GSE56081 sample groups. The threshold for gene differential expression screening was set at an adjusted *p*-value of less than 0.05 and a log_2_ fold change (FC) greater than 1 or less than −1. Heat maps were created using the heatmap package in R. In order to explore the potential features of the DEGs, we utilized the clusterProfiler program package to conduct GO and KEGG enrichment analyses. Additionally, we generated a volcano plot of the DEGs utilizing the OmicStudio tools (https://www.omicstudio.cn) (Lyu et al., 2023).

### 2.3 Ferroptosis-related genes and Venn diagramming

To identify ferroptosis-related genes, we downloaded 64 genes from the M39768. gmt gene set using the Gene Set Enrichment Analysis (GSEA) tool available at http://www.gsea-msigdb.org/gsea/index.jsp. Furthermore, we used Venn diagrams available at http://bioinformatics. psb.ugent.be/webtools/Venn/to map the DEGs associated with ferroptosis.

### 2.4 Weighted gene correlation network analysis (WGCNA)

The WGCNA was conducted using the “WGCNA” package according to the protocol in R software (Langfelder and Horvath, 2008). In our study, we first conducted gene expression profiling and then excluded genes with a standard deviation of 0 across all samples. We then utilized the WGCNA goodSamplesGenes approach to eliminate any outlier genes and samples. Additionally, we employed WGCNA to construct a scale-free co-expression network using β as a soft-thresholding parameter to highlight strong correlations between genes and penalize weak correlations. After determining the power of 10, the adjacency matrix was transformed into a topological overlap matrix (TOM) to measure the network connectivity of a gene. This is achieved by summing the adjacency values of a gene with all other genes in the network and dividing it by the ratio of the network gene. The corresponding dissimilarity can be calculated as (1-TOM) (Langfelder et al., 2008).

### 2.5 Immune infiltration analyses

The xCell algorithm is a deconvolution algorithm that estimates the abundance of immune cells in a sample using RNA sequencing (RNA-seq) data. It uses linear support vector regression for the expression matrix of immune cell subtypes (Aran et al., 2017). We utilized the xCell algorithm in R software to analyze 67 immune cell types in patients with different immune patterns, employing box plots. The proportions of immune cells in each group were evaluated via the Wilcoxon rank-sum test. A statistically significant difference was considered when *p* < 0.05. Furthermore, we predicted the abundance of immune cells using the immune cell abundance identifier (ImmuCellAI) (http://bioinfo.life.hust.edu.cn/web/ImmuCellAI) (Miao et al., 2020). ImmuCellAI is a web-based tool that utilizes a gene set signature method to estimate the abundance of 24 immune cell types. These cell types include B cells, natural killer cells, monocytes, macrophages, neutrophils, dendritic cells, and 18 T cell subtypes. The tool does this by analyzing expression data.

### 2.6 Upstream regulator network construction

The X2K tool (https://amp.pharm.mssm.edu/X2K/) was utilized to calculate the regulatory correlations between transcription factors (TFs), kinases, and intermediate proteins based on hypergeometric *p*-values (Clarke et al., 2018). Doma-Gen (https://ai.citexs.com) is a comprehensive database of human genes and diseases that aims to analyze human genes from multiple perspectives. Its goal is to explore the molecular mechanisms of diseases and their translational applications, and to contribute significantly to precision medicine approaches.

### 2.7 Enrichment analysis and epigenetic regulation of ferroptosis-related genes

In this study, we utilized various online genomic analysis platforms such as Gene Set Cancer Analysis (GSCALite: http://bioinfo.life.hust.edu.cn/web/GSCALite/) and STRING (https://string-db.org/) to generate a PPI network of ferroptosis-related genes (Liu et al., 2018). The PPI network of ferroptosis-related genes was generated using the STRING website (Shen et al., 2022).

### 2.8 Cell culture

Intervertebral disc tissues were collected from rats, and the NP tissues were extracted and cut into small pieces. The samples were then placed in 15-mL centrifuge tubes containing 2 mg/mL collagenase Ⅱ complete medium and incubated for 4–6 h at 37°C in a 5% CO_2_ incubator until the tissue was fully dissolved. The digested tissues were transferred to DMEM/F12 medium (DMEM/F12; HyClone, Logan, UT, United States of America) containing 10% fetal bovine serum (FBS; Gibco, Shanghai, China) and 1% penicillin/streptomycin. On the sixth day, cell growth was observed using an inverted-phase contrast microscope. The 10% FBS solution was replaced every 3 days, and the cells were subcultured once they reached 90% confluency. For further analysis, recombinant human IL-1β (obtained from PeproTech in New Jersey, United States of America) was dissolved in water and diluted to a concentration of 10 ng/m in the cell culture medium.

### 2.9 RNA extraction, reverse transcription, and quantitative PCR

RNA extraction was carried out using the TRIzol reagent (Beyotime, Shanghai, China) following the manufacturer’s instructions. Quantitative real-time polymerase chain reaction (qPCR) was performed using the SYBR Green Master Mix (Yeasen, China) and the 7500 Real-Time Polymerase Chain Reaction System for 40 cycles of amplification. Relative gene expression was determined using the 2^−ΔΔCT^ method. The primer sequences used for qPCR analysis can be found in Supplementary Table S1.

### 2.10 Western blot analysis

Protein lysates were obtained using RIPA buffer supplemented with protease and phosphatase inhibitors. The protein concentrations were determined using the bicinchoninic acid (BCA) protein assay kit (#ZJ101, Epizyme, China). Western blot analysis was conducted following previously described methods (Chen et al., 2020). The study utilized several primary antibodies, including anti-NCOA4 (#A5695, 1:1,000), anti-GPX4 (#A21440, 1:1,000), anti-PCBP1 (#A1044, 1:1,000), anti-collagen II (#A19308, 1:1,000), anti-p16 (#A0262, 1:1,000), and anti-β-actin (#AC026, 1:1,000), all of which were purchased from ABclonal.

### 2.11 Histological and immunohistochemistry (IHC) staining

In 2022, NP tissues from individuals who underwent spinal surgery at Shanghai East Hospital Affiliated to Tongji University were collected. The information on clinical specimens and imaging is provided in Supplementary Table S1. The degenerated NP samples were stored in liquid nitrogen to prevent RNA degradation. Additionally, all disc samples were fixed in 4% paraformaldehyde, embedded in paraffin, and sectioned. Hematoxylin–eosin (H&E) staining was utilized to observe histological modifications in degenerated intervertebral disc specimens, using a light microscope. Furthermore, the protein expression of P21, collagen II, NCOA4, PCBP1, and GPX4 in human disc degeneration tissues was examined through IHC and immunofluorescence (IF) staining.

### 2.12 Senescence-associated β-galactosidase (SA-β-gal) activity analysis

To model replicative senescence in NPCs, we adopted the approach of Jeong et al. (2014). Senescence-associated β-galactosidase (SA-β-gal) activity was assessed using a staining kit (Beyotime, China) in accordance with the manufacturer’s instructions to detect the presence of SA-β-gal in the cells.

### 2.13 Statistical analysis

The data were analyzed using R and GraphPad Prism software. Spearman’s correlation coefficient was used to evaluate the correlation between continuous variables. Differences among three or more groups were assessed using a one-way evaluation of variance (ANOVA), while differences between two groups were assessed using a t-test. A significance level of *p* < 0.05 was used to determine statistical significance.

## 3 Results

### 3.1 Identification of DEGs and enrichment analyses

**FIGURE 1:**
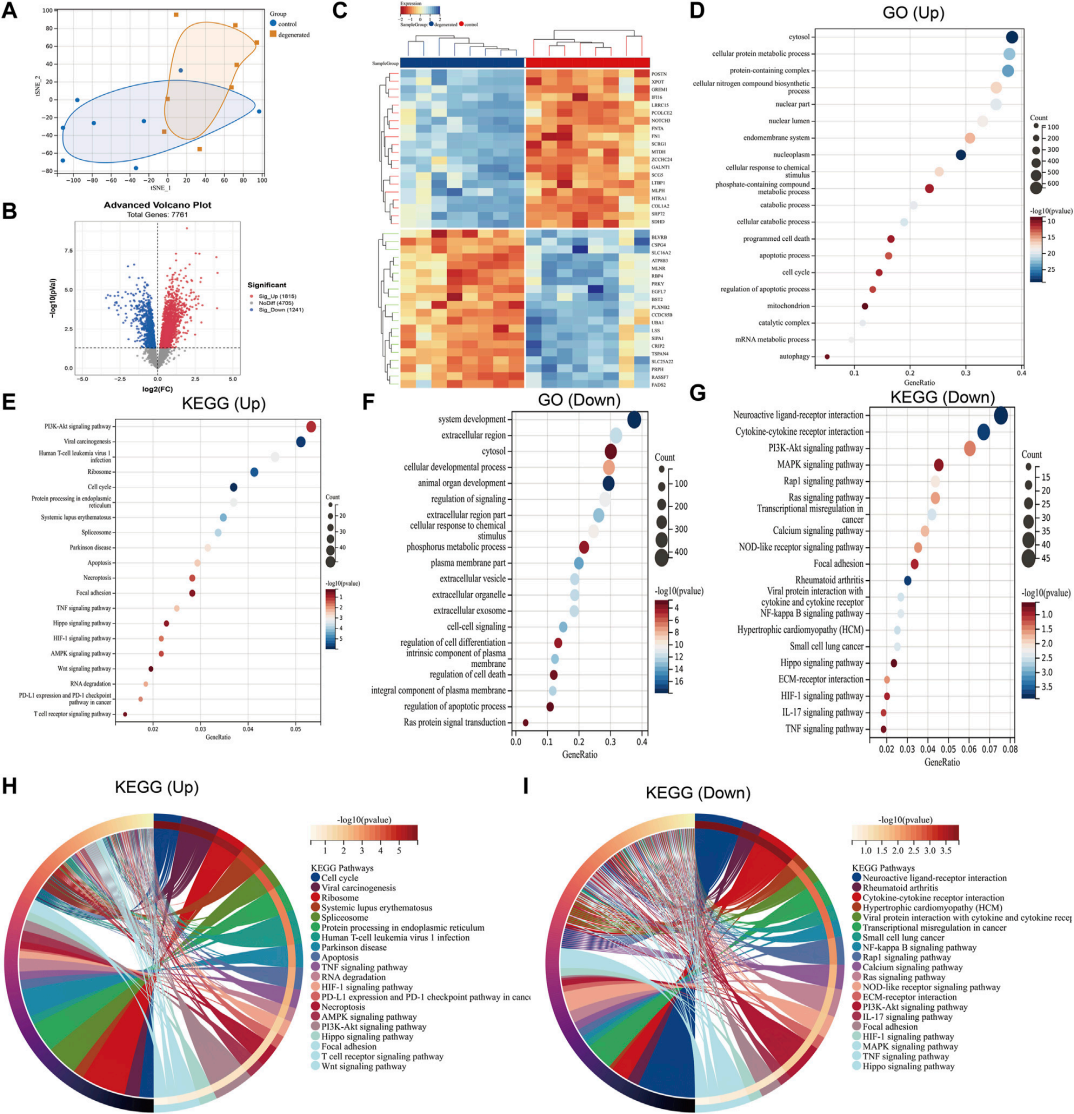
Differential mapping analysis of IVDD **(A)** t-distributed stochastic neighbor embedding (t-SNE) depicting the whole profile of the two datasets. **(B)** Volcano plots of DEGs. **(C)** Thermal plots of DEGs. **(D–G)** Bubble diagram represents GO and KEGG enrichment analysis of DEGs. **(H–I)** KEGG chord plot showing the top 20 biological processes.

The distribution of the samples was visualized using t-distributed stochastic neighbor embedding (t-SNE) (Figure 1A). After screening, a total of 3,056 differentially expressed genes were detected, with 1,815 genes upregulated and 1,241 genes downregulated (|log_2_ FC|>1 and *p* < 0.05). To better visualize the DEG profile, a volcano plot was created (Figure 1B). Heat maps were generated to display the top 20 upregulated and top 20 downregulated DEGs (Figure 1C). Enriched GO terms for the upregulated DEGs included cytosol, cellular protein metabolic process, programmed cell death, apoptotic process, cell cycle, and autophagy (Figure 1D). In addition to GO terms, the enriched KEGG pathways included the cell cycle; apoptosis; necroptosis; and the PI3K-Akt, Hippo, and HIF-1 signaling pathways (Figure 1E). The downregulated DEGs were found to be associated with several enriched GO terms, including system development, extracellular region, cellular developmental process, regulation of signaling, and phosphorus metabolic process (Figure 1F), while enriched KEGG pathways included neuroactive ligand–receptor interaction and the PI3K-Akt, MAPK, and Ras signaling pathways (Figure 1G). The KEGG chord plot is used to illustrate the enriched upregulated and downregulated genes for unique KEGG pathways (Figure 1H, I).

### 3.2 WGCNA and identification of critical modules

**FIGURE 2:**
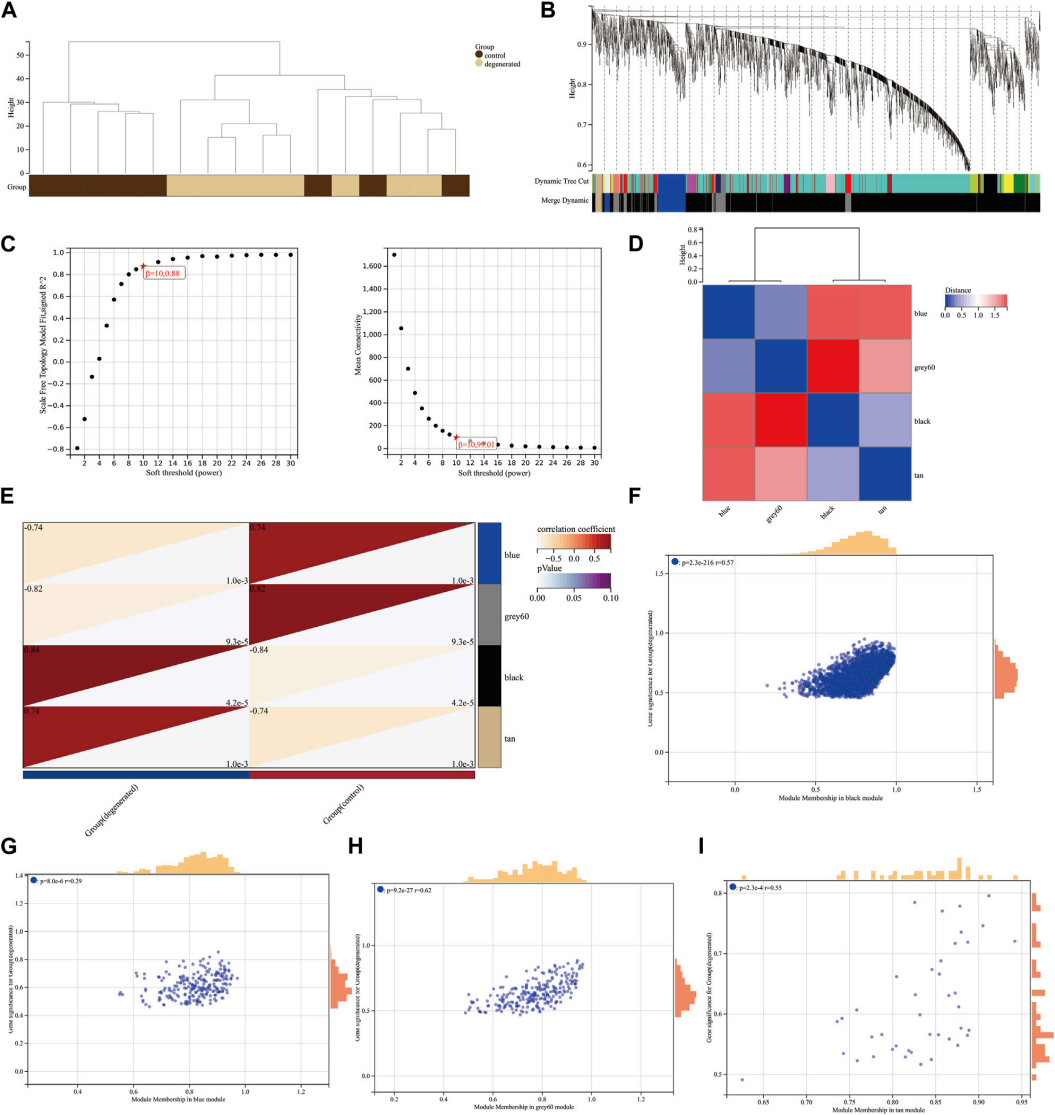
Construction of weighted co-expression network and identification of significant modules **(A)** Genetic tree diagram. **(B)** Clustering dendrogram of the clinical data from 16 IVDD samples. **(C)** Analysis of the scale-free ft index and mean connectivity for various soft-thresholding powers beta. **(D)** Module feature vector clustering heat map. **(E)** Heat map of the correlation between module eigengenes (MEs) and clinical characteristics of IVDD patients. Each cell contains the correlation coefficient and *p*-value. **(F–I)** Scatter plots of the GS score and MM for genes in the four modules.

We utilized the WGCNA package along with clinical data to construct a network comprising 3,056 DEGs (Figure 2A). Cluster evaluation was conducted on the eight samples to assess data quality. A height cut-off value of 200 was set to identify and eliminate any outliers that were not viable, before proceeding with further evaluation (Figure 2B). The first-rate soft threshold for this model was determined to be 10 using the pick soft threshold function. This resulted in an R2 value of 0.88 and a mean connectivity of 99.01 (Figure 2C). The differentially expressed genes that showed similar expression patterns were grouped into four co-expression modules: blue, gray60, black, and tan (Figure 2D). The eigengenes of the black module showed a complete positive correlation with IVDD, with a correlation coefficient of 0.84 and a *p*-value of 4.2 x 10^−5^. On the other hand, the eigengenes of the blue module exhibited a strong negative correlation with IVDD, with a correlation coefficient of −0.74 and a *p*-value of 1.0 x 10^−3^ (Figure 2E). The results suggest that the black module may play a role in the development of IVDD, while the blue module may have a protective effect against it. As a result, further analysis was conducted on the hub genes of both modules. The correlation between MM and GS ratings was found to be somewhat significant (Figures 2F–I).

### 3.3 Immune microenvironment characteristics of IVDD

**FIGURE 3:**
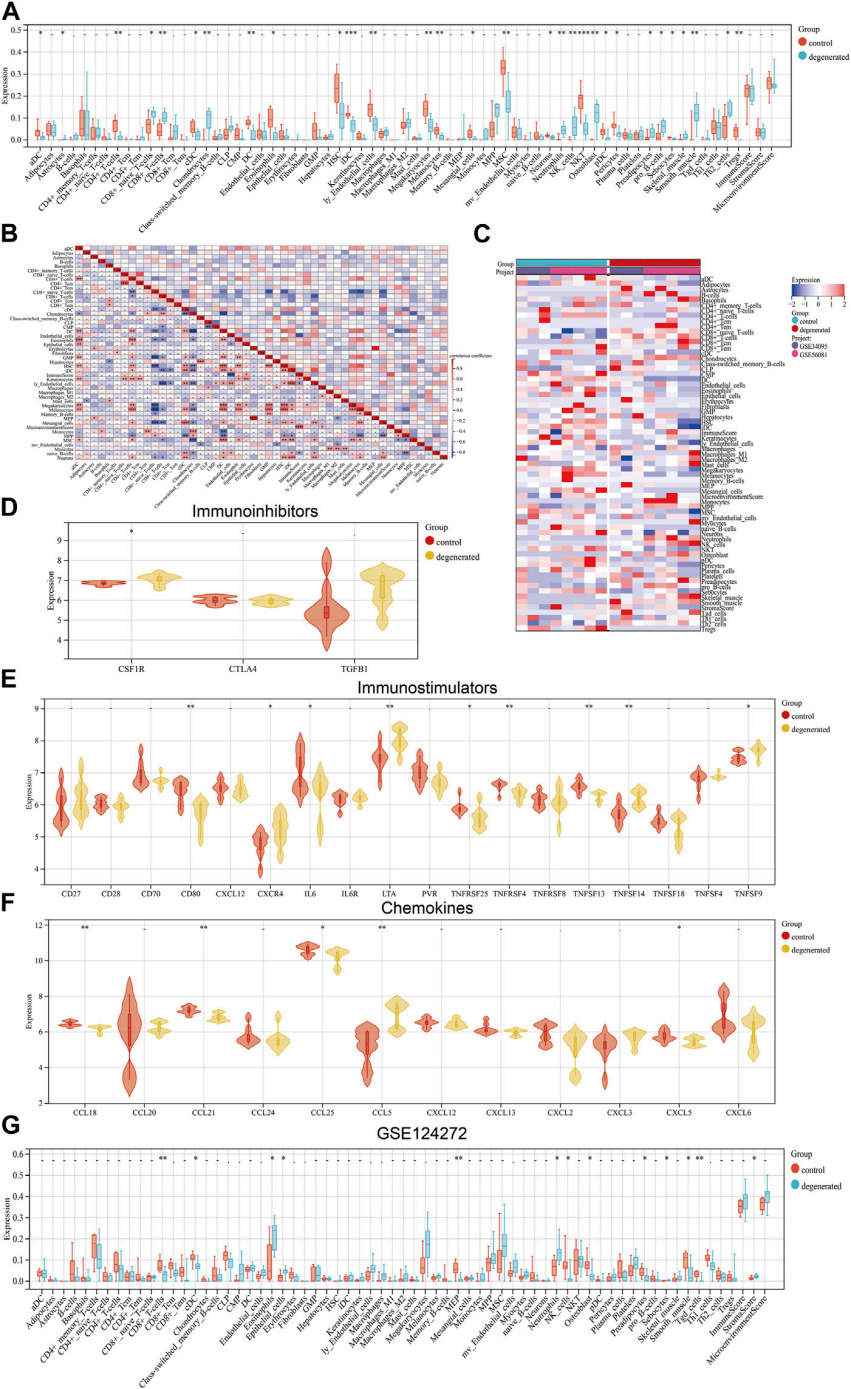
Landscape of immune infiltration in IVDD **(A)** Violin plot of the immune cell proportions. **(B)** Correlation matrix of immune cell proportions: * represents the significance of the correlation. **(C)** Heat map of the proportions of immune cell types. **(D–F)** Violin plot of the immune factors. **(G)** Violin plot of the immune cell proportions in GSE124272.

The immune system is not allowed to operate at full capacity in the *in vitro* diagnostic organ, which is known as an immune-privileged organ. This is because the immune system’s constant state of being in a state of immune privilege helps maintain the organ’s homeostasis (Sun et al., 2020). The development of IVDD leads to an increase in inflammatory cytokine levels within the IVD tissues, as well as an increase in the degradation of aggrecan and collagen. Additionally, the phenotype of IVDD cells undergoes changes (Francisco et al., 2022). Chemokines are released from degenerated IVD tissues which support the infiltration of immune cells, thus amplifying the inflammatory cascade (Rand et al., 1997; Le Maitre et al., 2007b). In order to assess the immunological signatures in the degenerated intervertebral disc, we utilized both immune cell infiltration and immune checkpoint molecule expression levels. To gain a deeper understanding of the immune microenvironment in the degenerated IVD, we employed the xCell algorithm to analyze the specific types of immune cells that infiltrated the IVDD tissue. Results from the examination of 67 types of immune cells showed that the levels of astrocytes, CD8+_naive_T-cells, CD8+_T-cells, chondrocytes, neutrophils, NK_cells, osteoblasts, preadipocytes, pro_B-cells, and Th2_cells were significantly higher in degenerated IVDD (*p* < 0.05) (Figures 3A–C). The percentages of 24 immune cell types were estimated in both the control and IVDD samples using ImmuCellAI and can be observed in the histogram (Supplementary Figure S1A). The immune cell infiltration of control and IVDD samples is depicted in a boxplot (Supplementary Figure S1B). The results of the study showed that the IVDD samples had higher percentages of B cells, macrophages, NK cells, CD8^+^ T cells, CD8+_naive_T-cells, Tex cells, and Tcm cells, while the proportions of DC cells and Tem cells were lower than those of the control group. Previous studies have also confirmed that CD68^+^ macrophages, neutrophils, and both CD4^+^ and CD8^+^ T cells can infiltrate the herniated disc (Kokubo et al., 2008). A relationship between IFN-γ and IVDD has been established. IFN-γ is secreted with the help of TH1 cells, NK cells, and macrophages (Schroder et al., 2004). The study illustrated in Figures 3D–F investigated the expression levels of various immune checkpoint molecules in IVDD. The results showed that CSF1R, CXCR4, LTA, TNFSF14, TNFSF9, and CCL5 were expressed at higher levels in the IVDD group than in the control group. Furthermore, the GSE124272 dataset revealed that immune cell infiltration in IVDD predominantly concerned neutrophils (Figure 3G). Research has shown that degenerated intervertebral disc cells have the ability to produce chemokines, including CCL2, CCL5, CSF2, and CXCL8. These chemokines have been found to stimulate the infiltration and activation of T cells, B cells, and macrophages, which can lead to increased degeneration (Khan et al., 2020). In general, the data have been noticeably clear between the training and validation sets.

### 3.4 Identification of DEGs and ferroptosis-related genes

**FIGURE 4:**
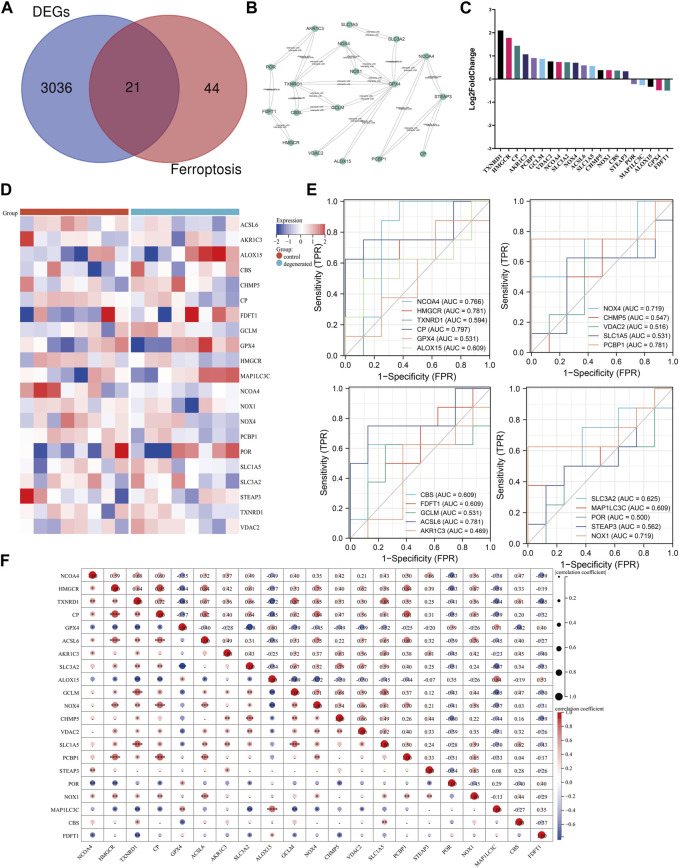
Identification of candidate genes. **(A)** Venn diagram between ferroptosis-related genes and DEGs. **(B)** Ferroptosis-related gene PPI network. **(C)** Heat map of correlation analysis between the 21 screened genes. **(D)** Clustered heat map of ferroptosis-related genes. **(E)** ROC curve evaluation of candidate genes. **(F)** Correlation matrix of ferroptosis-related genes: * represents the significance of correlation, and the number represents the correlation level. (*p < 0.05, **p < 0.01, and ***p < 0.001).

Research has demonstrated a correlation between ferroptosis and various health conditions, including neurodegenerative diseases, cancer, stroke, and cerebral ischemia–reperfusion injury (Chen W. et al., 2021; Wang Y. et al., 2022). Recent research has begun to explore the role of ferroptosis in IVDD. Zhang et al. discovered a significant enrichment of genes related to ferroptosis in the mild-IVDD group, suggesting the involvement of ferroptosis in the early stages of the disease (Zhang et al., 2021). This finding aligns with that of previous research on neurodegenerative diseases and kidney damage (Devos et al., 2014; Zhang P. et al., 2020). We performed an overlap analysis of the ferroptosis-related gene set with the DEGs and selected 21 genes that were common to both sets for further analysis. Out of these 21 genes, 16 genes were found to be upregulated, while five genes were downregulated. The Venn diagram evaluation confirmed that all 21 genes were ferroptosis-related genes (Figure 4A). The PPI network of ferroptosis-related genes was generated using the STRING database and visualized using Cytoscape software. An analysis of the network revealed that TXNRD1 can interact with NOX4, GPX4, GCLM, CBSL, and AKR1C3. Additionally, GPX4 can interact with SLC3A2, NCOA4, ALOX15, VDAC2, GCLM, TXNRD1, NOX4, and NOX1 (Figure 4B). Figures 4C, D display the expression of ferroptosis-related genes using a heat map and bar layout, respectively. To confirm the effectiveness and sensitivity of the chosen candidates, we conducted an evaluation using the receiver operating characteristic (ROC) curve. The results showed that the area under the ROC curve (AUC) values ranged from 0.5 to 0.8, indicating their validity (Figure 4E). After analyzing the 21 genes, it was found that there were strong positive associations between the expression levels of the HMGCR gene and those of CP, ACSL6, NOX4, and PCBP1 (Figure 4F). To validate the results, a separate dataset independent of the microarray datasets GSE41883, GSE27494, GSE23130, and GSE70362 was used. The expression levels of CP and STEAP3 were found to be significantly upregulated in aged NPCs and IVDD tissues. We conducted a comparison of the differentially expressed genes between severe (grades IV and V) and mild (grades I and III) degeneration in order to identify candidate genes that are related to the progression of IVDD. Our analysis revealed that the following genes may be potential candidates: PCBP1, CHMP5, CBS, HMGCR, NCOA4, VDAC2, STEAP3, and SLC1A5. The consistency of the results between the training and validation sets was also observed (Supplementary Figure S2). Further analysis was conducted on the correlations between differentially expressed genes related to ferroptosis and immune cells. The study found that the gene expression levels of HMGCR, CP, and PCBP1 were positively correlated with various immune cell types, such as CD4^+^ T cells, cDCs, and naive B cells (Supplementary Figure S3).

### 3.5 Upstream regulatory network of ferroptosis-related genes

**FIGURE 5:**
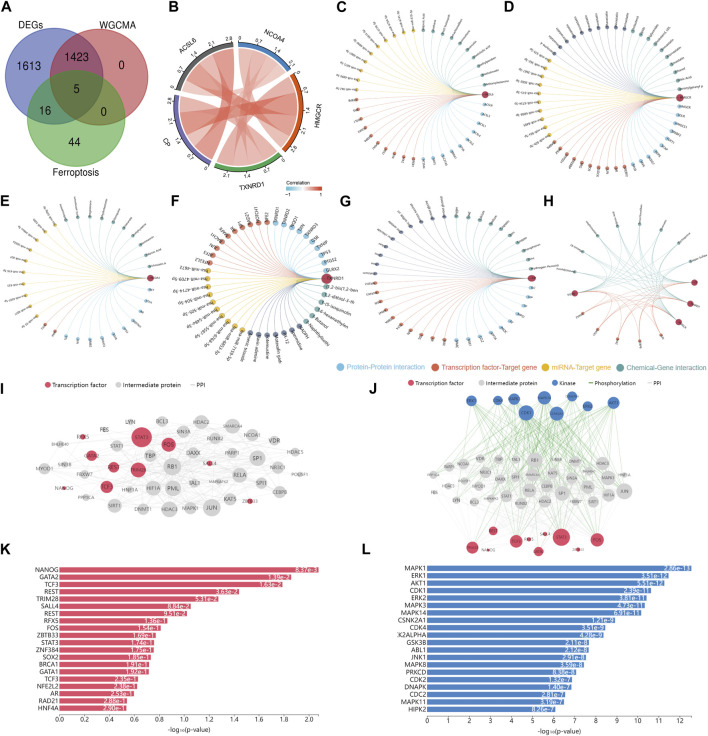
Upstream regulatory network prediction according to the downregulated DEGs **(A)** Five hub genes were obtained by taking the intersections of DEGs, WGCNA genes, and ferroptosis-related genes. **(B)** Interactions between characteristic genes at the molecular level. **(C–H)** Potential microRNAs and transcription factors (TFs) that regulate ferroptosis-related genes. **(I)** Predicted TFs and intermediate protein network diagram. **(J)** Regulatory network diagram according to the prediction of the downregulated DEGs. The node sizes are scaled proportional to the corresponding degree. **(K)** Kinases and **(L)** TFs according to the predictions of the downregulated DEGs.

After analyzing the intersections between the differentially expressed genes, genes obtained from weighted gene co-expression network analysis, and genes related to ferroptosis, a total of five hub genes were identified (Figures 5A, B). To explore potential features of ferroptosis-related genes, we utilized the Doma-Gen (https://ai.citexs.com) database to predict microRNAs (miRNAs) and transcription factors that may modify these genes (Figures 5C–H). Activation transcription factor 3 (ATF3) plays a crucial role in regulating NCOA4 and TXNRD1. Numerous studies have demonstrated that ATF3 promotes both ferroptosis and apoptosis in various diseases. In HT1080 cells induced by erastin, ATF3 directly binds to the SLC7A11 promoter and inhibits SLC7A11 expression, thereby promoting ferroptosis (Wang et al., 2020). Our study focuses on the involvement of TFs, kinases, and intermediate proteins (Figures 5I, J). We anticipate that NANOG, GATA2, TCF3, REST, TRIM28, SALL4, REST, RFX5, and other upstream TFs have a significant role in this process (Figure 5K). Furthermore, we found that MAPK1, ERK1, AKT1, CDK1, ERK2, MAPK3, MAPK14, and CSNK2A1 are the most strongly correlated kinases (Figure 5L).

### 3.6 Somatic mutations, copy number variation (CNV), and epigenetic regulation of the ferroptosis-related genes

**FIGURE 6:**
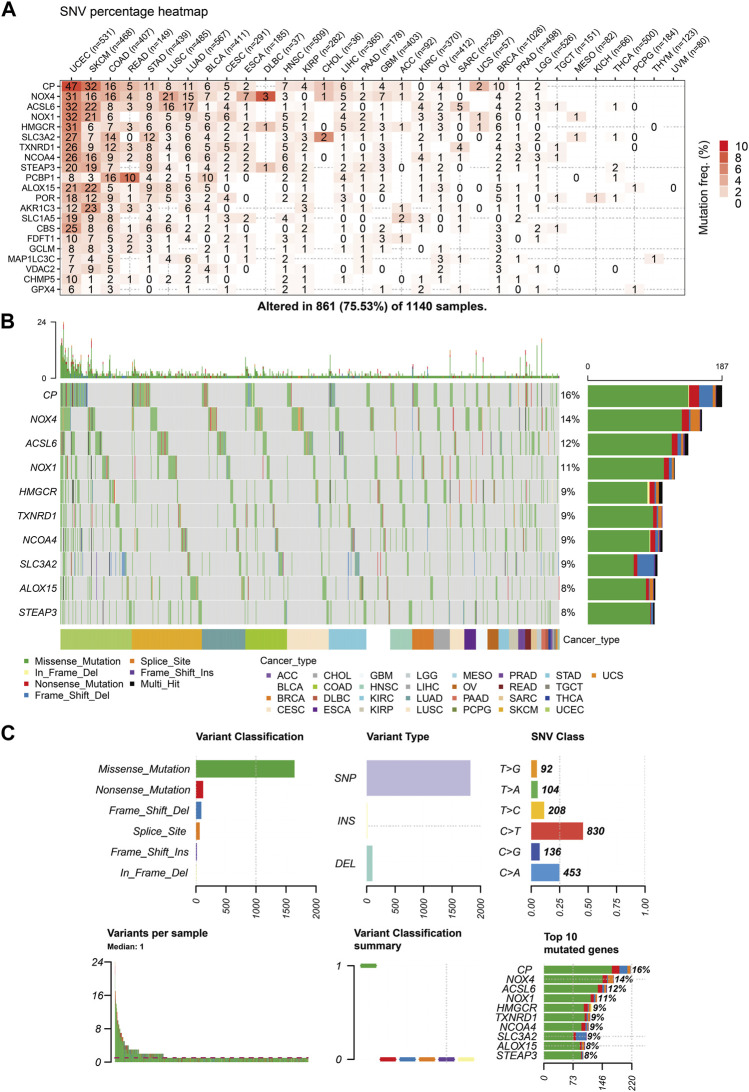
Single-nucleotide variation (SNV) frequency and variant types of the ferroptosis-related genes **(A)** Heat map showing the SNV frequencies of 21 ferroptosis-related genes across different cancer types. **(B)** Waterfall plot depicting the SNVs of the top ten mutated genes among the ferroptosis-related genes in the specific cancers. **(C)** SNV classes of the ferroptosis-related genes in the pan-cancer analysis.

**FIGURE 7:**
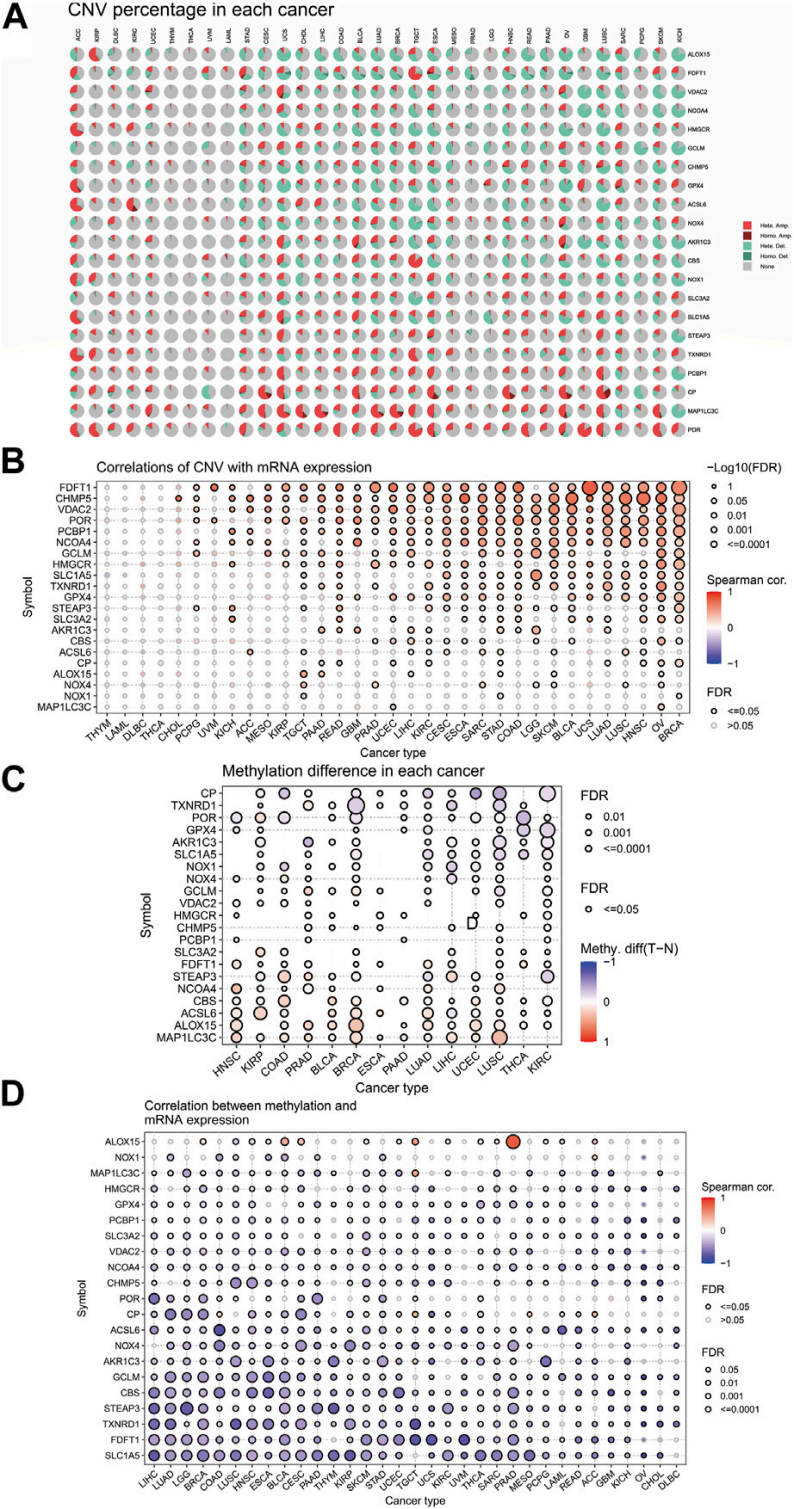
Associations between ferroptosis-related genes, genomic features, and expression. **(A)** Copy number variation (CNV) pie chart showing the proportions of different types of CNVs in each gene across diverse cancer types. **(B)** Correlations between CNV and mRNA expression levels. **(C)** Profile of correlations between methylation and mRNA expression levels. **(D)** Methylation differences between tumor and normal samples of ferroptosis-related genes in cancers. Blue dots represent negative correlations, and red dots represent positive correlations. The sizes of the dots represent significance. Hete Amp: heterozygous amplification; Hete Del: heterozygous deletion; Homo Amp: homozygous amplification; Homo Del: homozygous deletion; and None: no CNV.

The similarities between aging and certain cancers are striking, as many of the hallmarks of aging, such as genomic instability, epigenetic alterations, and chronic inflammation, closely resemble specific cancer characteristics. These shared features can be thought of as common ‘meta-signatures.’ However, there are also aspects of aging, such as telomere attrition and stem cell exhaustion, which may actually inhibit the development of tumors, making them ‘antagonistic features’ (Lopez-Otin et al., 2023). We conducted an analysis of The Cancer Genome Atlas (TCGA) database research data to assess the frequency and variation of ferroptosis-related genes. Figure 6A illustrates that among the various cancers studied, the frequency of single-nucleotide variation (SNV) ranged from 1% to 47% in UCEC, SKCM, and COAD. The ferroptosis-related genes examined had an SNV frequency of 75.53% (861 out of 1,140 tumors). The missense mutation was found to be the most common type of ferroptosis-related gene mutation through SNV evaluation. The top 10 mutant genes, CP, NOX4, ACSL6, NOX1, HMGCR, TXNRD1, NCOA4, SLC3A2, ALOX15, and STEAP3, had mutation percentages ranging from 8% to 16%. The SNV frequency of ferroptosis-related genes was examined in UCEC, SKCM, LUAD, and COAD (Figures 6B, C). In this study, the CNV data of ferroptosis-related genes were analyzed in the TCGA database to identify any relevant CNV alterations. The resulting pie chart distribution confirmed that the main CNVs in these genes were heterozygous amplification and deletion. Further evaluation of the CNV proportions revealed that the heterozygous amplification of FDFT1, VDAC2, HMGCR, GPX4, ACSL6, CBS, NOX1, SLC1A5, TXNRD1, and POR was higher than 25% in ACC (*p* < 0.05, Figure 7A). Subsequent analysis confirmed a positive correlation between CNV and mRNA expression levels for most genes. However, there were a few exceptions where CNV exhibited a negative correlation with gene expression, namely, SLC1A5 in TGCT, NOX4 in LGG, and CP in READ (*p* < 0.05, Figure 7B). In this study, we conducted further analysis on the methylation levels of ferroptosis-related genes to investigate their epigenetic regulatory mechanisms. Our findings show that the methylation of these genes is highly heterogeneous in certain tumors. Specifically, we observed a greater number of hypermethylated genes than hypomethylated genes in HNSC, KIRP, COAD, PRAD, BLCA, BRCA, LUAD, LIHC, UCEC, and LUSC. In KIRC, it was observed that there were more hypomethylated genes than hypermethylated ones. However, FDFT1, STEAP3, NCOA4, CBS, ACSL6, ALOX15, RBL2, and MAP1LC3C were found to be hypermethylated in most cancers, with a *p*-value less than 0.05 (Figure 7C). In our study, we investigated the correlation between methylation patterns and mRNA expression levels. Our findings revealed a negative association between the methylation level and expression levels of most genes. However, we observed a positive correlation between the methylation of ALOX15 in BRCA, ESCA, BLCA, CESC, UCEC, TGCT, SARC, PRAD, PCPG, and ACC and gene expression levels (*p* < 0.05, Figure 7D). Our study delved deeper into the analysis of ferroptosis-related genes from a genomic perspective, focusing on the frequency and mutation types, with the aim of investigating the role of epigenetic modifications in the progression of IVDD.

### 3.7 Bioinformatics result validation

**FIGURE 8:**
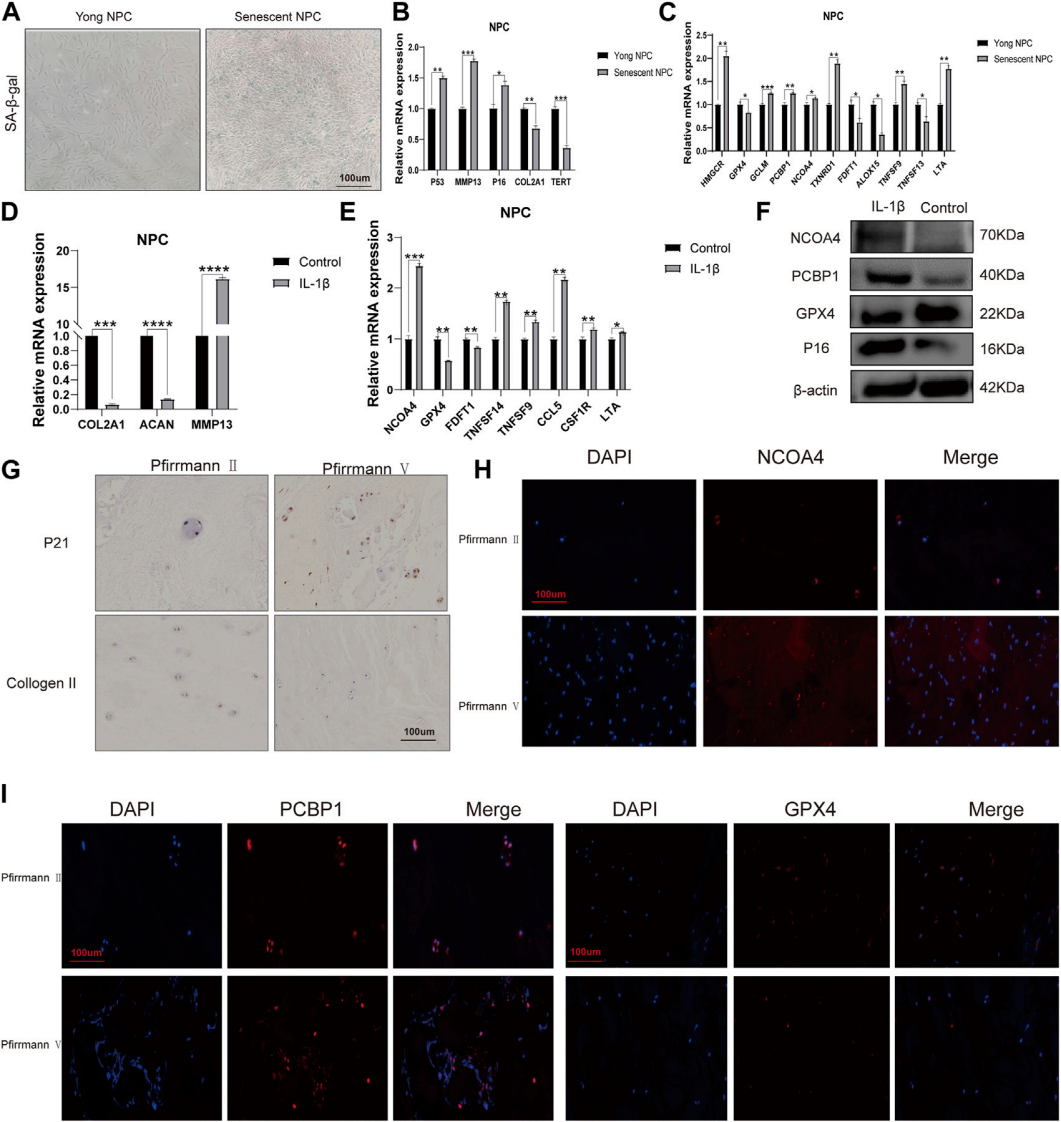
mRNA expression levels of candidate genes **(A)** Representative images of nucleus pulposus cell (NPC) senescence. **(B)** Relative mRNA expression levels of aging markers in senescent NPCs. **(C)** Relative mRNA expression levels of ferroptosis-related genes and immune-related markers in senescent NPCs. **(D)** Relative mRNA expression levels of aging markers in NPCs treated with IL-1β. **(E)** Relative mRNA expression levels of ferroptosis-related genes and immune-related markers in NPCs treated with IL-1β. **(F)** Western blot analysis of NCOA4, PCBP1, and GPX4 protein expression in NPCs treated with IL-1β. **(G)** Immunohistochemical staining of p21 and collagen II in the disc samples. **(H, I)** Immunofluorescence staining of NCOA4, PCBP1, and GPX4 in human disc degeneration tissues. (*p < 0.05, **p < 0.01, and ***p < 0.001).

First, we determined nucleus pulposus cell senescence by SA-β-gal staining experiments (Figure 8A). The study found significant increases in mRNA expression levels of P53, P16, and MMP13 in the old NPC group, with MMP13 showing the largest difference. Conversely, the mRNA expression levels of COL2A1 were found to be decreased in the same group. The human telomerase reverse transcriptase (TERT) gene is responsible for encoding telomerase, which plays a crucial role in maintaining genome integrity by lengthening telomeres. Due to certain obstacles, the process of telomere lengthening is incomplete, resulting in telomere shortening in cells after each mitosis cycle. This eventually leads to replicative senescence (Hayflick, 1965; Nandakumar and Cech, 2013). Our results demonstrate that TERT expression levels are extensively decreased in senescent NPCs (Figure 8B). To validate the results obtained from bioinformatics analysis, we conducted qPCR experiments. Our findings confirmed that the mRNA expression levels of HMGCR, GCLM, PCBP1, NCOA4, and TXNRD1 were significantly higher in the senescent NPC group, while the mRNA expression levels of GPX4, FDFT1, and ALOX15 were decreased. Our hypothesis was supported by these consistent findings. Further analysis of immune-related markers revealed a significant decrease in TNFSF13 expression levels in senescent NPCs, while TNFSF9 and LTA expression levels were markedly increased (Figure 8C). To establish an *in vitro* model of NPC degeneration, IL-1β was administered, and the expression of senescence-associated markers was validated through qPCR analysis (Figure 8D). Upon confirmation of cell senescence, it was found that the mRNA expression levels of NCOA4, TNFSF14, TNFSF9, CCL5, CSF1R, and LTA were significantly elevated, while the mRNA expression levels of GPX4 and FDFT1 were considerably reduced in the IL-1β group (Figure 8E). The study found that IL-1β treatment resulted in increased protein expression levels of NCOA4 and PCBP1, while the expression of GPX4 protein was reduced in NPCs. This was confirmed through Western blot analysis (Figure 8F). The IHC staining evaluation indicated that the protein expression levels of collagen II were reduced while those of p21 were elevated, with an increasing grade of human disc degeneration (Figure 8G). The immunofluorescence results indicate a significant increase in the expression levels of NCOA4 and PCBP1 proteins, while the expression level of GPX4 protein was decreased in human intervertebral disc degeneration tissues (Figures 8H, I). To confirm the infiltration of immune cells in IVDD, we conducted immunohistochemical staining using specific markers for immune cells. Our study revealed a significant increase in the expression of CD8A in naive T cells, CCR7 in memory CD4^+^ cells, GZMB in NK cells, and CD163 and CD45 in macrophages in the presence of severe disc tissue degeneration (Supplementary Figure S4). The aforementioned results further affirm that the data mining and bioinformatics analysis procedures are reliable and have promising research value.

## 4 Discussion

The pathological mechanism of IVDD is not yet fully understood, and both surgical and conservative treatment methods can have negative side effects. As a result, researchers are exploring new early prognostic strategies, including the identification of relevant genetic elements (Kepler et al., 2013; Li et al., 2022). In recent years, the combination of WGCNA and microarray technology has been widely utilized in the study of a variety of diseases, including different types of cancers (Langfelder and Horvath, 2008; Li et al., 2021). WGCNA, in conjunction with other analytical methods, can help connect pattern traits with gene expression profiles. Thus, a thorough and comprehensive analysis is necessary to identify potential biomarkers for IVDD. Previous studies have highlighted the importance of the intervertebral disc as an immune-privileged organ, indicating a strong association between immune infiltration and IVD tissues (Sun et al., 2020). This study aimed to investigate gene-level variations in the tissues of IVDD patients using a combination of DEGs and WGCNA evaluation to identify potential biomarkers. The study screened a total of 3,056 DEGs and conducted GO and KEGG analyses, which revealed that these DEGs are primarily involved in the cell cycle; apoptosis; necroptosis; and the PI3K-Akt, Hippo, and HIF-1 signaling pathways. The differentially expressed genes identified in this study are found to be involved in various cellular processes such as cell development, signaling regulation, phosphorus metabolism, and pathways related to PD-L1 expression, PD-1 checkpoints, T cell receptors, IL-17, TNF, and NOD-like receptors. The findings of this study indicate that the DEGs are predominantly associated with immunity. The outcomes of WGCNA confirmed the generation of four exclusive clustered co-expression modules. Among these modules, the black module was found to potentially promote disc degeneration, while the blue module was found to possibly shield the disc from IVDD. Our results were tested using a module-trait and module-eigengene adjacency heat map.

Numerous studies have shown that the intervertebral disc is an immuno-privileged organ, meaning that it is not recognized as foreign by the immune system. However, immune infiltration can significantly impact the progression of intervertebral disc degeneration (Sun et al., 2020). For several decades, researchers have made significant efforts to elucidate the connection between intervertebral disc degeneration and immune cells (Risbud and Shapiro, 2014). Exposure to NP may influence the autoimmune response through its association with T cells, B cells, and neutrophils (Wang and Samartzis, 2014). In animal models, Geiss et al. discovered that subcutaneous injection of autologous NP led to increased activation of T and B cells (Geiss et al., 2009). Further investigation and discussion are necessary to understand the nuances of the IVDD immune microenvironment. Our research indicates that the progression of IVDD is significantly linked to the balance of various immune cells and other cell types, including CD8+_naive_T-cells, CD8+_T-cells, chondrocytes, neutrophils, NK_cells, osteoblasts, preadipocytes, pro_B-cells, and Th2_ cells. Neutrophils, a type of white blood cell, are essential components of the innate immune system (Castanheira and Kubes, 2019). Our study found that neutrophil levels were increased in IVDD patients, which is consistent with the findings of previous research (Bozkurt et al., 2019). This reinforces the importance of neutrophil infiltration in mediating the improvement of IVDD. Additionally, our study observed higher expression levels of immune checkpoint molecules, including CSF1R, CXCR4, LTA, TNFSF14, TNFSF13, and CCL5, in IVDD tissues. Our data indicate that the expression of TNFSF13 is significantly reduced in senescent NPCs. Conversely, the expression levels of TNFSF9 and LTA are significantly increased.

Ferroptosis is a type of cell death that is caused by iron-dependent lipid peroxidation. It occurs in various biological contexts, including development, aging, immunity, and cancer (Stockwell, 2022). In their study, Zhang et al. revealed that ferroptosis plays a role in the development of intervertebral disc degeneration. As a result, targeting ferroptosis could potentially serve as a novel therapeutic approach for treating IVDD (Zhang et al., 2021). Research has demonstrated that ferroptosis, induced by oxidative stress and iron overload, can contribute to the calcification of cartilage endplates and hasten the progression of intervertebral disc degeneration (Wang W. et al., 2022). Studies have shown that iron deficiency plays a role in the development of IVDD by triggering apoptosis (Yang et al., 2021). Although the role of ferroptosis in IVDD progression remains unclear, this study provides further analysis of its potential involvement in the pathogenesis of IVDD. Through the identification of 21 ferroptosis-related genes, it was found that 16 were upregulated and five were downregulated. Notably, TXNRD1, HMGCR, and CP were highly expressed in IVDD tissues, indicating their potential significance in the development of IVDD. TXNRD1 is a crucial regulator that plays a significant role in the ferroptosis of chronic myeloid leukemia cells (Liu et al., 2021). Ceruloplasmin (CP) is a glycoprotein that plays a crucial role in iron homeostasis (Vashchenko and MacGillivray, 2013; Yang et al., 2022). *In vitro* cultured NPCs treated with IL-1 showed high expression of TXNRD1 and CP.

MAP1LC3C was found to be significantly downregulated in aged NPCs and IVDD tissues. Furthermore, the expression levels of PCBP1, CHMP5, CBS, HMGCR, NCOA4, VDAC2, STEAP3, and SLC1A5 were observed to be higher in severe (grades IV and V) degeneration when compared to mild (grades I and III) degeneration. According to our experimental results, the mRNA expression levels of HMGCR, GCLM, PCBP1, NCOA4, and TXNRD1 were found to be extensively increased in the senescent NPC group. However, the mRNA expression levels of GPX4, FDFT1, and ALOX15 were decreased. Furthermore, our IHC and IF assays showed that the protein expression levels of NCOA4 and PCBP1 were increased, while GPX4 protein expression was decreased in both human IVDD tissues and senescent NPCs.

To gain a better understanding of the frequency of mutations and the genes related to ferroptosis, we analyzed TCGA research data. Our analysis of SNVs revealed that missense mutations were the most common type of mutation in ferroptosis-related genes. Additionally, we found that CP and NOX4 were the two most frequently mutated genes. Furthermore, our genetic evaluation showed that ferroptosis-related genes are frequently affected by CNVs. Our study found a positive correlation between mRNA expression and CNV, specifically for FDFT1, UCS, and CHMP5 in HNSC. We also confirmed a significant correlation between CNV and expression levels of ferroptosis-related genes, suggesting that CNV can affect the expression of clock genes and contribute to IVDD. This article discusses the role of ferroptosis and immune infiltration in intervertebral disc degeneration, using bioinformatics analyses to provide new insights. The findings could have implications for understanding and treating this common spinal condition.

## Figures and Tables

**Table:** 

**MD**	Mild degeneration
**SD**	Severe degeneration
**NPS**	Nucleus pulposus sample
**NPC**	Nucleus pulposus cell
**ACC**	Adrenocortical carcinoma
**BLCA**	Bladder urothelial carcinoma
**BRCA**	Breast invasive carcinoma
**CESC**	Cervical squamous cell carcinoma and endocervical adenocarcinoma
**CHOL**	Cholangiocarcinoma
**COAD**	Colon adenocarcinoma
**DLBCL**	Lymphoid Neoplasm Diffuse large B-cell lymphoma
**ESCA**	Esophageal carcinoma
**FPPP**	FFPE pilot phase II
**GBM**	Glioblastoma multiforme
**HNSC**	Head and neck squamous cell carcinoma
**KICH**	Kidney chromophobe
**KIRC**	Kidney renal clear cell carcinoma
**KIRP**	Kidney renal papillary cell carcinoma
**LAML**	Acute myeloid leukemia
**LGG**	Brain Lower grade glioma
**LIHC**	Liver hepatocellular carcinoma
**LUAD**	Lung adenocarcinoma
**LUSC**	Lung squamous cell carcinoma
**MESO**	Mesothelioma
**OV**	Ovarian serous cystadenocarcinoma
**PAAD**	Pancreatic adenocarcinoma
**PCPG**	Pheochromocytoma and paraganglioma
**PRAD**	Prostate adenocarcinoma
**READ**	Rectum adenocarcinoma
**SARC**	Sarcoma
**STAD**	Stomach adenocarcinoma
**SKCM**	Skin cutaneous melanoma
**STES**	Stomach and esophageal carcinoma
**TGCT**	Testicular germ cell tumor
**THCA**	Thyroid carcinoma
**THYM**	Thymoma
**UCEC**	Uterine corpus endometrial carcinoma
**UCS**	Uterine carcinosarcoma
**UVM**	Uveal melanoma

## Data Availability

The original contributions presented in the study are included in the article/Supplementary Material, further inquiries can be directed to the corresponding author.
