# Supplementary material for: Role of ferroptosis and immune infiltration in intervertebral disc degeneration: novel insights from bioinformatics analyses
Source: Front Cell Dev Biol. 2023 Sep 6;11:1170758. doi: 10.3389/fcell.2023.1170758 (PMC10509768; doi:10.3389/fcell.2023.1170758)

**Information on human disc samples from 2 patients**

| **Human disc samples** | **Sex** | **Age** | **level** | **Grade** |
| --- | --- | --- | --- | --- |
| 1 | Female | 25 | L5/S1 | II |
| 2 | Female | 87 | L5/S1 | V |

**Human disc sample 1 (MRI)**


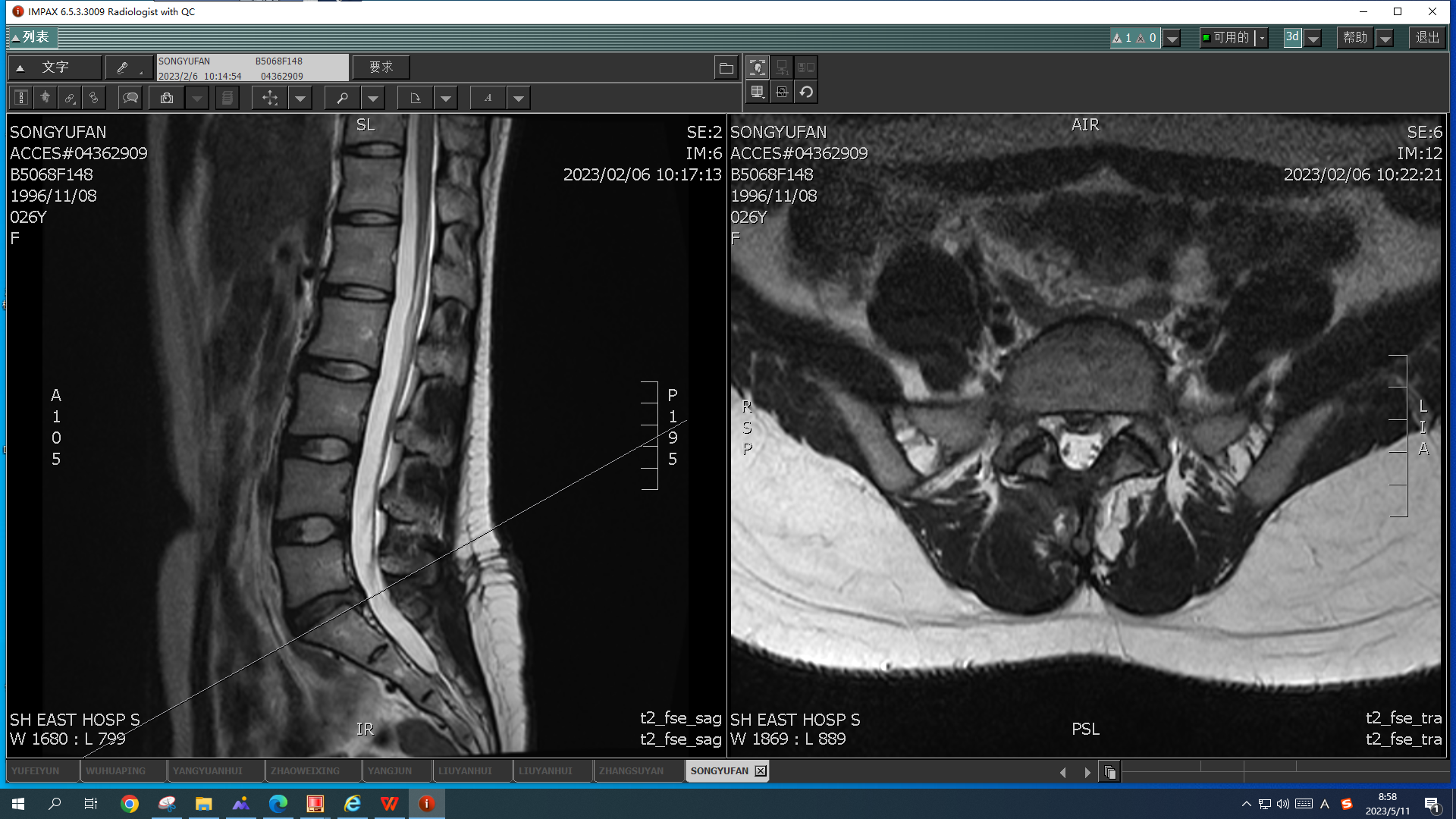


**Human disc sample 2 (MRI)**


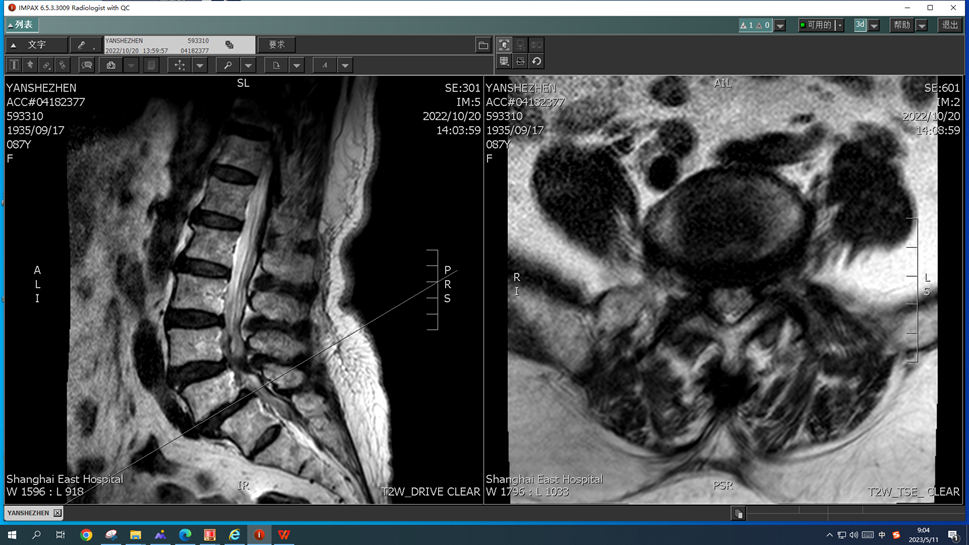

Supplement: Supplementary file 1 [file Table1.DOCX]
