# Supplementary figures and images for: Role of ferroptosis and immune infiltration in intervertebral disc degeneration: novel insights from bioinformatics analyses
Source: Front Cell Dev Biol. 2023 Sep 6;11:1170758. doi: 10.3389/fcell.2023.1170758 (PMC10509768; doi:10.3389/fcell.2023.1170758)

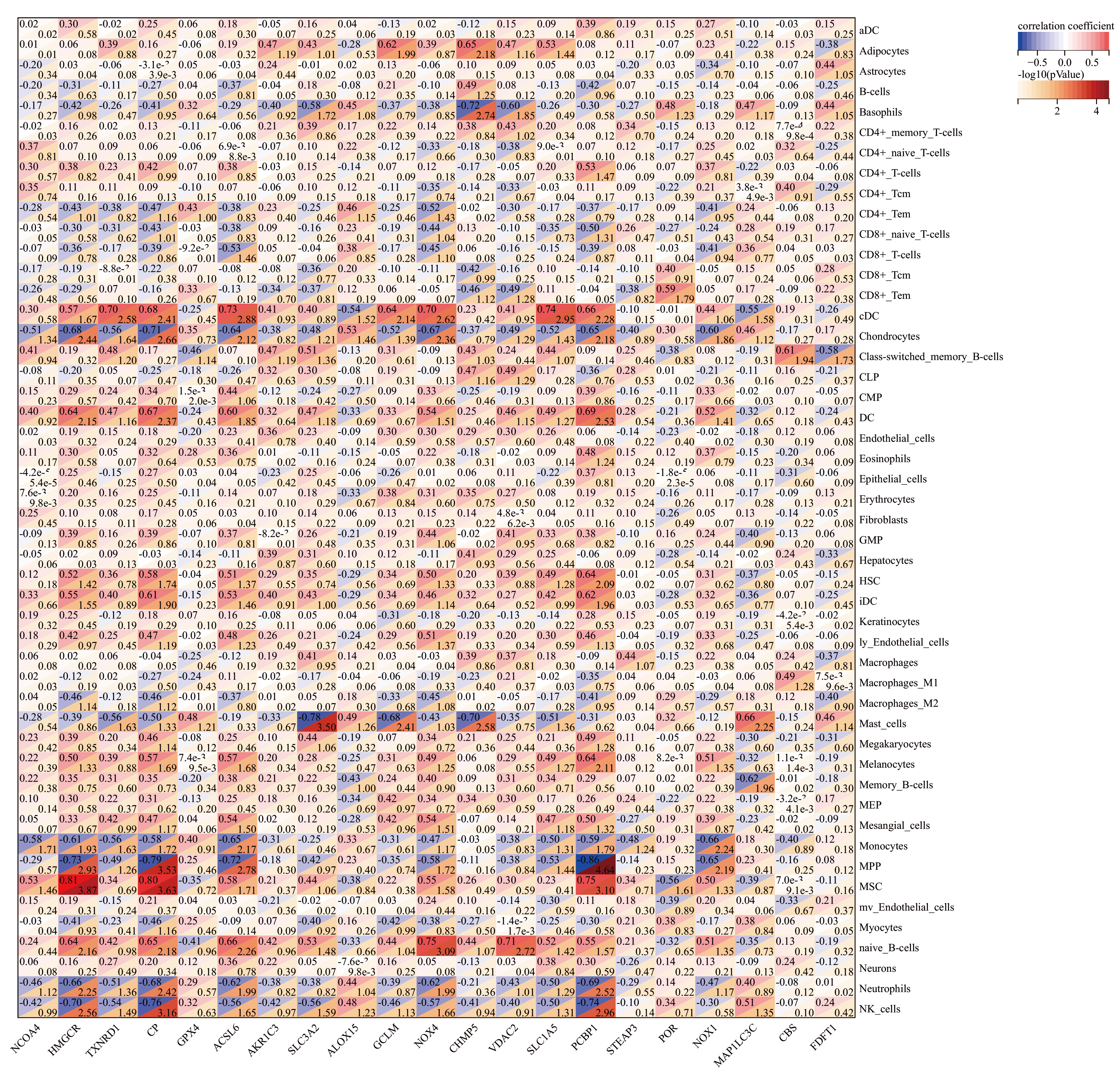

Supplement: Supplementary file 2 [file Image3.TIF]

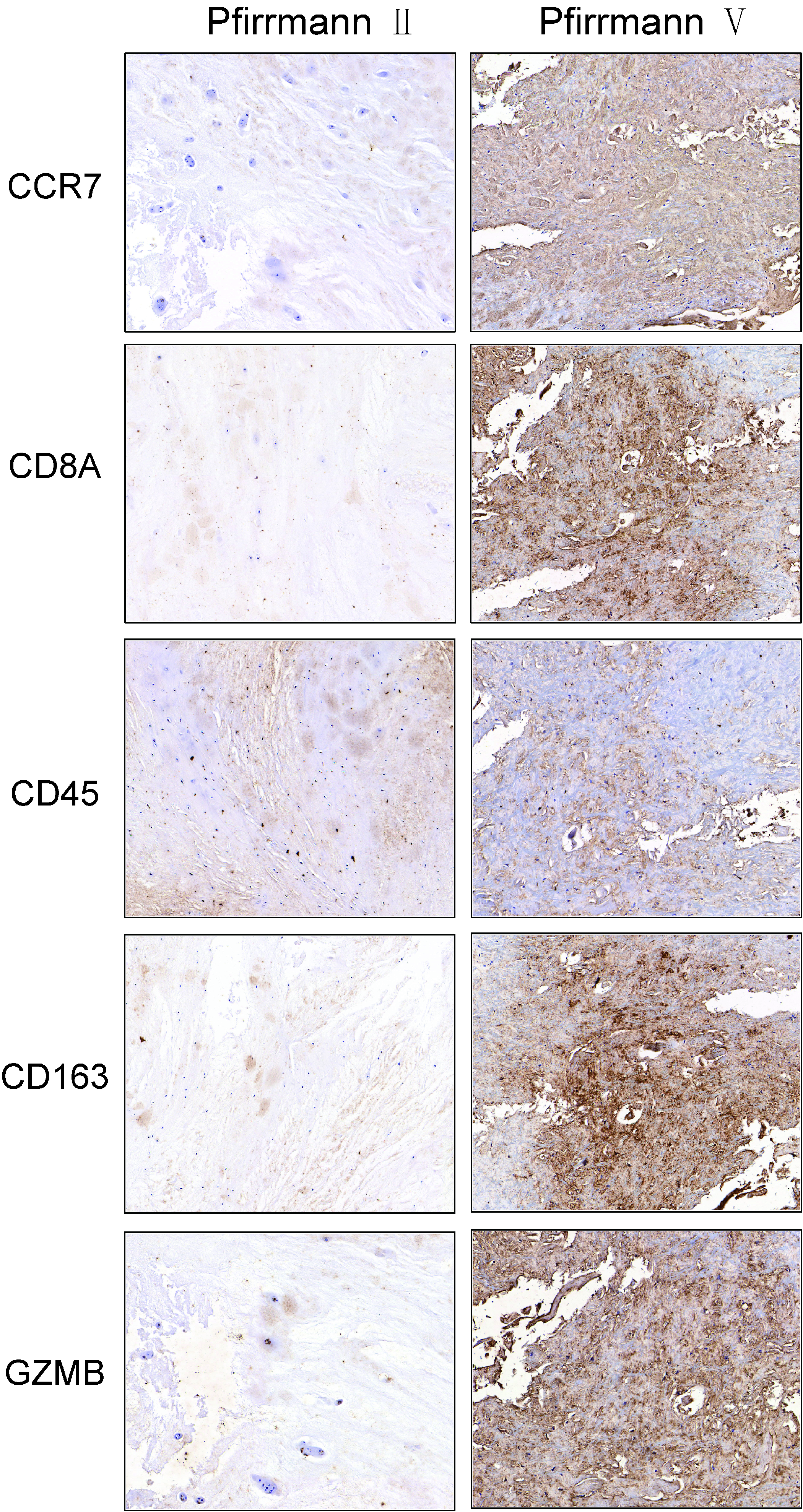

Supplement: Supplementary file 3 [file Image4.TIF]

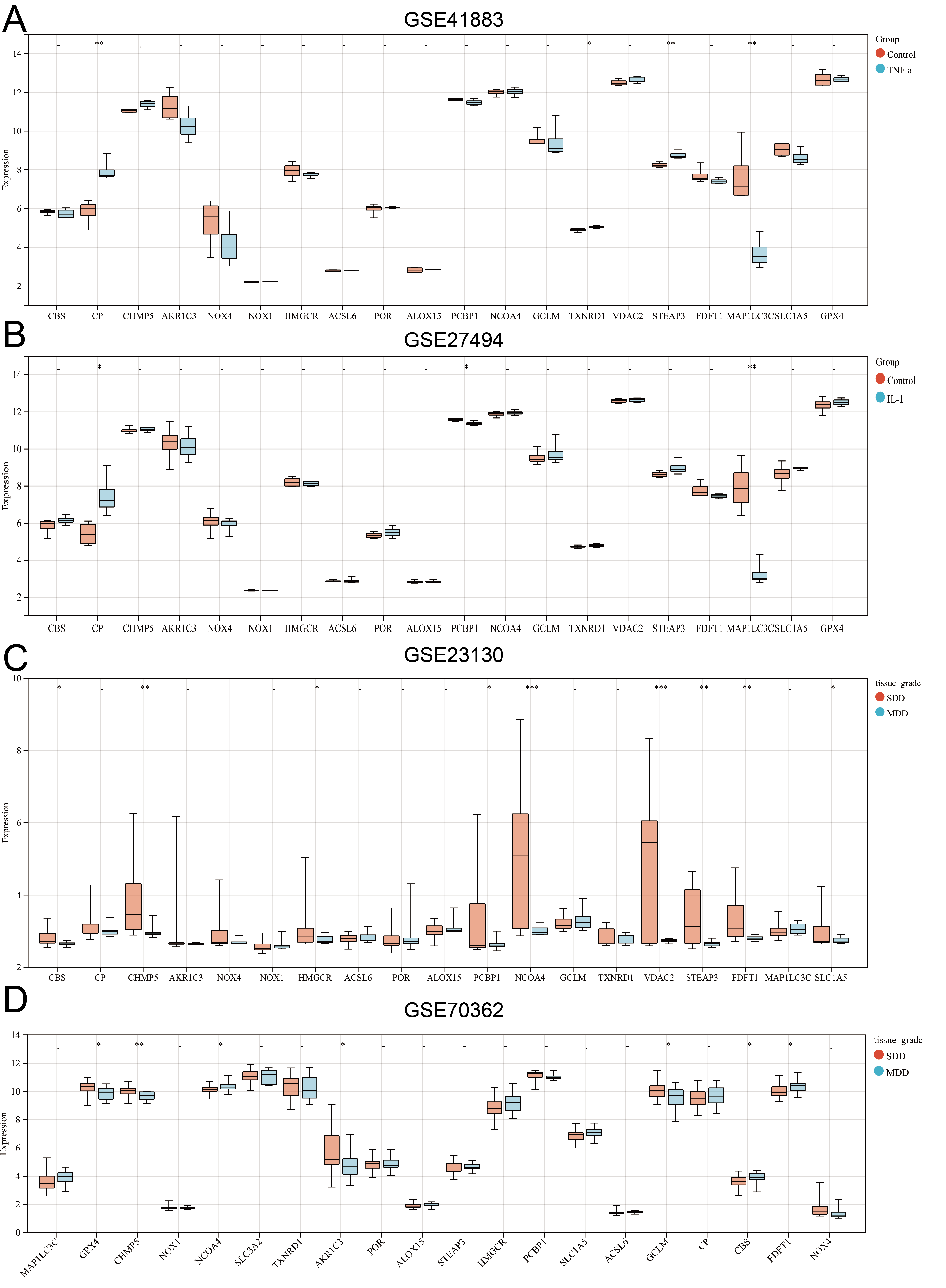

Supplement: Supplementary file 4 [file Image2.TIF]

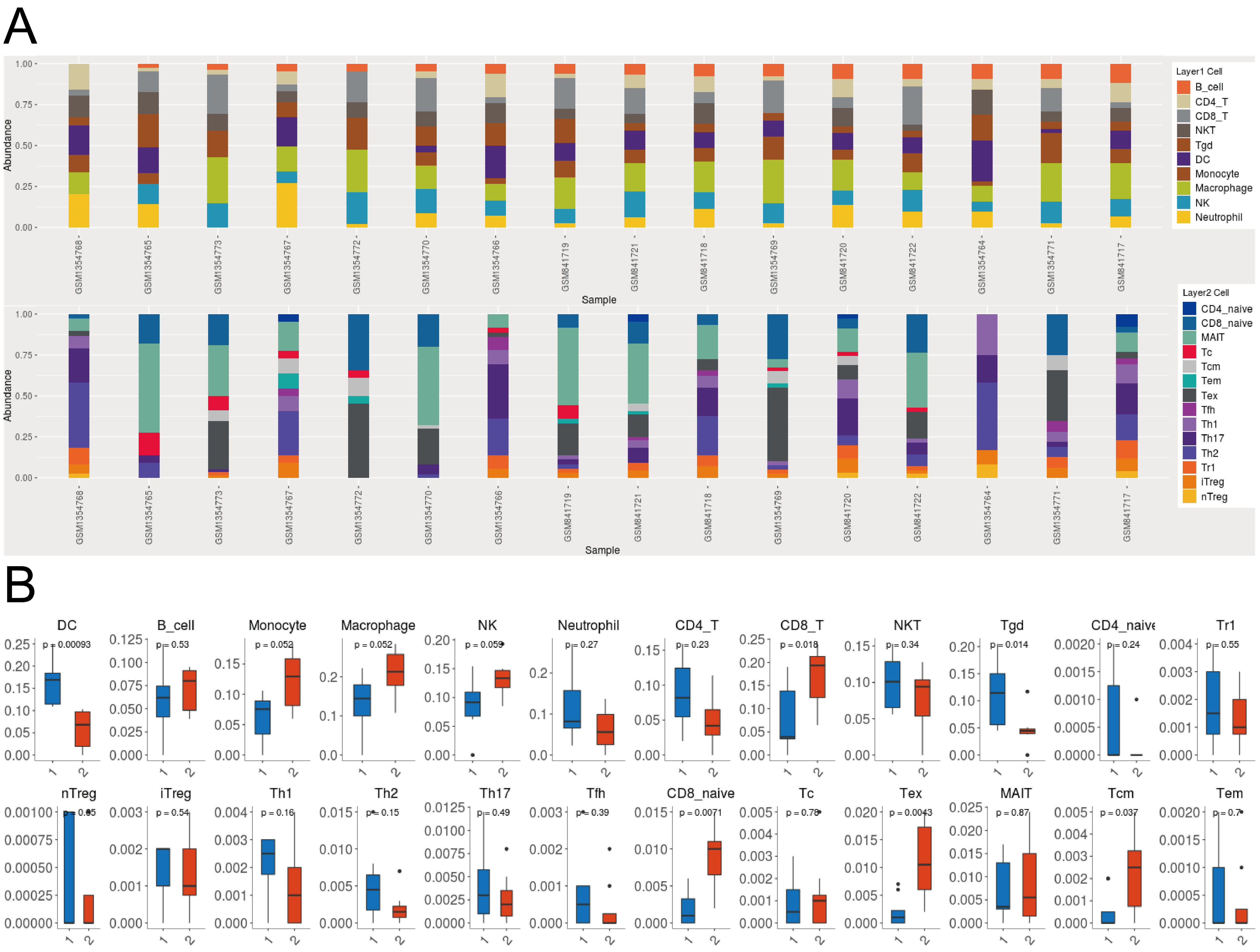

Supplement: Supplementary file 5 [file Image1.TIF]
